# Specific and number of comorbidities are associated with increased levels of temporomandibular pain intensity and duration

**DOI:** 10.1186/s10194-015-0528-2

**Published:** 2015-05-20

**Authors:** Haissam Dahan, Yoram Shir, Ana Velly, Paul Allison

**Affiliations:** Division of Oral Health and Society, Faculty of Dentistry, McGill University, Montreal, QC H3A 1G1 Canada; Alan Edwards Pain Management Unit, McGill University Health Centre, Montreal General Hospital, Montreal, QC H3G 1A4 Canada; Department of Dentistry, Jewish General Hospital, Montreal, QC H3T 1E2 Canada

**Keywords:** Temporomandibular disorder, Comorbidity, Pain duration, Pain intensity, Migraine, Chronic fatigue syndrome

## Abstract

**Background:**

Temporomandibular pain disorder (TMD) is a common pain condition in the face. People with TMD report multiple pain comorbidities. The presence of fibromyalgia and migraine in people with TMD is associated with an increase in TMD pain intensity and duration. However, data on the relationship between increasing number of pain comorbidities and TMD pain are rare. The aims of this study were: firstly to evaluate the extent to which increasing number of comorbidities is associated with increasing TMD pain intensity and duration; and secondly to evaluate the extent to which the presence of specific comorbidities is associated with increasing TMD pain intensity and duration.

**Methods:**

The sample included 180 people seeking TMD treatment at Boston and Montreal clinics. TMD was diagnosed using the Research Diagnostic Criteria for TMD. A Numerical Pain Rating Scale assessed TMD pain intensity and participants provided their TMD pain duration in a study questionnaire. The comorbidities of migraine, chronic fatigue syndrome, irritable bowel syndrome, interstitial cystitis and restless leg syndrome were diagnosed by 5 validated diagnostic questionnaires. The associations were analyzed by linear regression, controlling for confounders.

**Results:**

There was a positive association between the number of comorbidities present and TMD pain intensity (p < 0.01) and between the number of comorbidities present and TMD pain duration (p < 0.01). Also, the presence of migraine was positively associated with TMD pain intensity (p < 0.01) and the presence of chronic fatigue syndrome was positively associated with TMD pain intensity (p < 0.05) and with TMD pain duration (p < 0.01). When TMD patients were separated into groups, these associations did not change for the myofascial pain group, whereas in the non-myofascial pain group, the relationship between number of comorbidities and TMD pain duration was the only one still present.

**Conclusion:**

This study shows that the number of comorbidities is positively associated with TMD pain duration and intensity. The presence of specific conditions, such as migraine and chronic fatigue syndrome, is associated with an increase in TMD intensity and duration.

## Background

Temporomandibular pain disorder (TMD) is a common pain condition in the face, with a prevalence of 5–33 % [1, 2]. TMD includes pain in the temporomandibular joint, disc, and surrounding muscles [3]. People with TMD report multiple pain comorbidities [4, 5]. The presence of widespread pain is associated with onset and increase of TMD pain intensity, duration, and disability [6–10]. However, data on the relationship between TMD pain and multiple pain comorbidities that are not widespread is rare [11]. Widespread pain may not be a good indicator of multiple pain comorbidities since it tends to be more severe, longer lasting, and accompanied by decreased functionality and poorer general health [12]. Therefore, additional research is necessary to understand the relationship between multiple comorbidities and TMD pain intensity and duration.

Recent research in other pain conditions has revealed that some comorbidities interact with pain in more harmful ways. For example, having heart disease, depression, or anxiety increased the odds of having pain 5 years after total knee replacement by over 1.5 times, whereas having diabetes or renal disease did not [13]. In people with TMD, the presence of migraine and fibromyalgia is associated with an increased TMD pain and disability [6, 7, 14–17]. However, there are few data of whether other comorbidities are associated with TMD pain intensity or duration.

In addition, people with myofascial TMD (m-TMD) have been observed to have more severe and longer-lasting pain, and experience more dysfunction and disability when compared to people with non-myofascial TMD (j-TMD) [18]. However, it is unknown whether people with m-TMD exhibit differences in the relationships between number and specific comorbidities and TMD pain.

This study had three aims: Firstly, to describe the relationship between the number of comorbidities and TMD pain duration and intensity. We hypothesized that the number of comorbidities would be positively associated with levels of TMD pain intensity and duration. Secondly, we aimed to observe the association of five specific comorbidities and TMD pain duration and intensity. The presence of migraine was hypothesized to be associated with an increased TMD pain intensity and duration because of past studies indicating this relationship [15, 16]. No hypothesis was made for the other conditions since no prior data are available. Finally, we aimed to dichotomize participants into two TMD groups: m-TMD and j-TMD to test if the above associations remain the same for both TMD groups. We hypothesized that the same relationship would be observed in both groups.

## Methods

Ethical approval to perform the study was granted from the Institutional Review Boards of McGill University and the Massachusetts General Hospital before the start of data collection. A written informed consent form was obtained from all participants prior to joining the study. This cross-sectional multi-center study included patients with TMD treated in three clinics: The Division of Dentistry at the Montreal General Hospital, the Department of Oral and Maxillofacial Surgery and Orofacial Pain Clinic at the Massachusetts General Hospital. At the Massachusetts General Hospital patients that fulfilled the inclusion criteria were approached during routine clinic visits and were invited to participate. At the Montreal General Hospital, inactive patients of a TMD clinic that had since been closed that were deemed eligible by a chart-review were invited to join the study by a letter mailed to their last known address. Patients with chronic TMD, lasting for more than 6 months, were included in the study [19]. Excluded were patients with acute pain, other types of comorbid orofacial pain condition (neuropathic, burning mouth syndrome, atypical) and a history of TMJ surgery. Eligible participants who agreed to participate were diagnosed with TMD by a clinical exam using the Research Diagnostic Criteria (RDC/TMD) [20] by two experienced dentists. The Kappa score between the two clinicians was considered good (0.88, 95 % CI 0.65-1.00) [21]. The sample size was calculated for a linear regression model with an effect size between the outcome variables and predictor variable that is deemed as medium (F^2^ = 0.15), and a maximum number of predictors of 6 (the primary predictor variable, two known confounders like age and sex and three other potential confounders) [22]. A sample of 97 was deemed necessary for a significance level of 0.05 and power at 80 %.

### Data collection

The current study assessed the following variables: TMD pain intensity and duration as outcome variables, and presence and number of specific comorbidities as exposure variables. The comorbidities were migraine, chronic fatigue syndrome (CFS), irritable bowel syndrome (IBS), interstitial cystitis (IC), and restless leg syndrome (RLS), all more prevalent in people with TMD than the general population [4]. To assess the presence of specific comorbidities, each participant completed 5 validated diagnostic questionnaires: the ID Migraine [23], the Schedule of Fatigue and Anergia [24], the ROME III [25], the Pain, Urgency and Frequency Symptom Scale [26], and the Cambridge-Hopkins Restless Leg Syndrome Questionnaire-short form [27]. These questionnaires all have very good sensitivity (range 81 % to 93 %), specificity (range 70 % to 95 %), and positive predictive value (range 70 to 94 %). Participants also completed a study questionnaire that assessed sociodemographic information and history of depression and anxiety. To assess the outcome variables, the duration of TMD pain was assessed in the study questionnaire by asking, “How long have you had pain in the face?” Average current TMD pain intensity was assessed by the Numerical Pain Rating Scale [28] from 0 to 10.

Subjects were stratified into two TMD subgroups based on the presence of myofascial pain: m-TMD were participants who had myofascial pain, whether alone or in combination with other forms of TMD disorder; j-TMD were participants who were free of myofascial pain, but had other forms of TMD disorder. For the current study, m-TMD corresponded to the RDC/TMD group I (myofascial pain) alone, or in combination with group II (disc displacement) and/or group III (other joint conditions) and j-TMD corresponded to the RDC/TMD group II (disc displacement) and/or group III (other joint conditions).

### Data analysis

In order to reveal possible confounders, socio-demographic differences were compared between participants with and without comorbidities. Means and frequencies were used for continuous and categorical variables respectively. Differences in socio-demographic variables were tested using *T*-test for continuous variables and Chi-squared test for categorical variables. The selection of potential confounder included both a priori and empirical considerations. For empirical evidence, we included the covariates with a p-value equal to or less than 0.25 as confounders [29]. A test for co-linearity between the exposure variables was also completed using variance inflation factor (VIF) larger than 10 as evidence of co-linearity between two exposure variables.

To analyze the relationships between the number of comorbidities present and the two outcome variables (TMD pain intensity and duration) and between specific comorbidities and the two outcome variables (TMD pain intensity and duration), multivariate linear regression analyses were done taking into account the confounding variables. For each outcome variable, three models of regression were performed: 1) Crude Model with no confounding variables, 2) Complete Model including all potential confounders and 3) Simple Model including only confounders that met the change-of-estimate criterion set at 20 % [30].

Finally, we dichotomized the participants based on presence of myofascial pain (m-TMD and j-TMD) and re-ran the regression models to reveal the relationship of comorbidities and outcome variables based on TMD subgroups. Linear regression coefficient, the 95 % confidence intervals, p value and R^2^ values were estimated in all analyses. Statistical analyses were performed using Stata (version 10).

## Results

A total of 224 participants agreed to partake in the study, of which 209 participants were from the Massachusetts General Hospital and 15 were from the Montreal General Hospital. Forty-four subjects were excluded from the study: 31 for not fulfilling the inclusion criteria and 13 for not completing all questionnaires. A final sample of 180 people completed the study. The final sample of 180 participants completed the study, which 68 (37.8 %) had no comorbidities, 54 (30.0 %) had one comorbidity and 58 (32.2 %) had at least two comorbidities (Table 1). Participants with and without comorbidities differed in some of the socio-demographic characteristics: subjects with comorbidities displayed a more severe and longer duration of TMD pain and were more likely to have m-TMD, have a positive history of depression and/or anxiety and differ in work status.

**Table 1:** Socio-demographic characteristics of the study participants

Variables	No comorbidity	With comorbidity	P value
	(n = 68)	(n = 112)	
Age, mean years (SD)	44.77 (15.6)	41.59 (15.3)	0.18
Female gender, n (%)	52 (76.5)	97 (86.6)	0.08
Marital Status, n (%)			0.08
Single	17 (25.0)	46 (41.1)	
Married	43(63.2)	49 (43.8)	
Divorced	6 (8.8)	14 (12.5)	
Widowed	2 (2.9)	3 (2.7)	
M-TMD n (%)	37 (54.4)	84 (75.9)	<0.01
Pain intensity, mean (SD)	4.57 (2.7)	5.43 (2.3)	0.03
Pain Duration, mean years (SD)	4.50 (6.9)	7.44 (8.5)	0.02
Positive history of depression and/or anxiety, n (%)	30 (44.1)	70(62.5)	0.02
Work Status, n (%)			0.02
Unemployed	11 (16.7)	33 (29.5)	
On disability	1 (1.5)	11 (9.8)	
Part-time	17 (25.0)	15 (13.4)	
Full-time	39 (57.3)	53 (47.3)	
Education Status, n (%)			0.85
Did not finish high school	4 (5.8)	5 (4.5)	
Finished high school	14 (20.6)	22 (19.6)	
Attended some college	50 (73.5)	84 (75.0)	
Comorbidities, n (%)			
0	68 (100)		
1		54 (48.2)	
2		30 (26.8)	
3		19 (17.0)	
4+		9 (8.0)	
Type of comorbidity, n (%)			
Migraine	N/A	83 (46.1)	
IBS		51 (28.3)	
RLS		30 (16.7)	
CFS		26 (14.4)	
IC		22 (12.2)	

The covariate analysis for outcome variable, TMD pain duration, revealed that the confounders were sex, TMD group, marital status, work status and psychological history. Age was not found to be significant as a confounder but was included in the regression model due to the overwhelming evidence from previous research of the association with the chosen variables [31]. The covariate analysis for outcome variable, TMD pain intensity, revealed that the confounders were TMD group, work status and psychological history. Sex and age were included in the regression model from previous research of the association with our variables [31]. The test for co-linearity revealed that the exposure variable - the number of comorbidities, was highly correlated with the five tested comorbidities (VIF = 94). This result is not surprising, since the presence or absence of the five comorbidities in a participant defined his/her number of comorbidities (0 to 5).

**Fig. 1:**
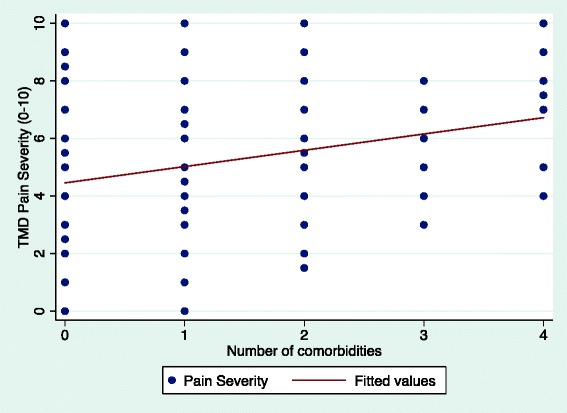
The association between TMD pain intensity (0–10) and the number of comorbidities in all TMD participants (n = 180)

**Fig. 2:**
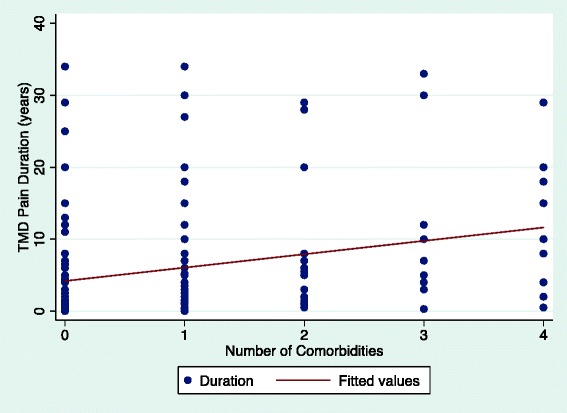
The association between TMD pain duration (years) and the number of comorbidities in all TMD participants (n = 180)

Our analyses demonstrated a positive association between the number of comorbidities and TMD pain intensity in a dose–response fashion (Fig. 1). A positive association was also found between the number of comorbidities and TMD pain duration (Fig. 2).

Tables 2 and 3 show the result of the linear regression analysis comparing TMD pain duration and intensity as outcome variables. After controlling for confounding variables there was a significant association between the number of comorbidities and TMD pain duration in all three regression models. As well, there was an association between the number of comorbidities and TMD pain intensity in all three models. The regression models of specific comorbidities indicated that the presence of CFS had the strongest association with both TMD pain duration and intensity. The presence of migraine was associated with a one-point increase in TMD pain intensity. The other comorbidities were not associated with either TMD pain intensity or duration.

**Table 2:** Results of multiple linear regression models comparing between comorbidities and TMD pain duration in the combined TMD group (n = 180)

Outcome variables	Exposure variables	Crude Model	Complete model	Simple model
		Coefficient (95 % CI)	Coefficient (95 % CI)	Coefficient (95 % CI)
		p value, r^2^ value	p value, r^2^ value	p value, r^2^ value
TMD duration (years)	Number of comorbidities	1.73 (0.78 – 2.68)	1.64 (0.62 – 2.65)	1.56 (0.59 – 2.53)
		p < 0.01, r^2^ = 0.06	p < 0.01, r^2^ = 0.13	p < 0.01, r^2^ = 0.13
	Migraine	1.56 (−0.83 – 3.95)	0.28 (−2.31 – 2.87)	0.01 (−2.47 – 2.49)
		p = 0.20, r^2^ = 0.01	p = 0.83, r^2^ = 0.17	p = 0.99, r^2^ = 0.16
	Chronic Fatigue Syndrome	6.58 (3.32 – 9.84)	6.03 (2.52 – 9.54)	5.84 (2.38 – 9.30)
		p < 0.01, r^2^ = 0.08	p < 0.01, r^2^ = 0.17	p < 0.01, r^2^ = 0.16
	Irritable Bowel Syndrome	3.03 (0.42 – 5.65)	2.24 (−0.41 – 4.90)	2.15 (−0.47 – 4.77)
		p = 0.02, r^2^ = 0.03	p = 0.10, r^2^ = 0.17	p = 0.11, r^2^ = 0.16
	Interstitial Cystitis	4.04 (0.44 – 7.64)	0.71 (−3.17 – 4.59)	0.78 (−3.03 – 4.59)
		p = 0.03, r^2^ = 0.03	p = 0.72, r^2^ = 0.17	p = 0.69, r^2^ = 0.16
	Restless Leg Syndrome	1.68 (−1.52 – 4.87)	−0.16 (−3.39 – 3.07)	−0.15 (−3.35 – 3.04)
		p = 0.30, r^2^ = 0.01	p = 0.92, r^2^ = 0.17	p = 0.92, r^2^ = 0.16

**Table 3:** Results of multiple linear regression models comparing between comorbidities and TMD pain intensity in the combined TMD group (n = 180)

Outcome variables	Exposure variables	Crude Model	Complete model	Simple model
		Coefficient (95 % CI)	Coefficient (95 % CI)	Coefficient (95 % CI)
		p value, r^2^ value	p value, r^2^ value	p value, r^2^ value
TMD intensity (0–10)	Number of comorbidities	0.57 (0.27 – 3.95)	0.46 (0.14 – 0.78)	0.46 (0.15 – 0.78)
		p < 0.01, r^2^ = 0.07	p < 0.01, r^2^ = 0.10	p < 0.01, r^2^ = 0.10
	Migraine	1.43 (0.71 – 2.15)	1.02 (0.20 – 1.84)	1.08 (0.30 – 1.87)
		p < 0.01, r^2^ = 0.08	p < 0.05, r^2^ = 0.15	p < 0.01, r^2^ = 0.14
	Chronic Fatigue Syndrome	1.85 (0.83 – 2.88)	1.07 (−0.03 – 2.19)	1.12 (0.03 – 2.22)
		p < 0.01, r^2^ = 0.07	p = 0.06, r^2^ = 0.15	p < 0.05, r^2^ = 0.14
	Irritable Bowel Syndrome	0.66 (−0.167 –1.48)	0.10 (−0.73 – 0.94)	0.10 (−0.73 – 0.93)
		p = 0.12, r^2^ = 0.01	p = 0.81, r^2^ = 0.15	p = 0.81, r^2^ = 0.14
	Interstitial Cystitis	1.40 (0.28 –2.52)	0.85 (−0.36 – 2.07)	0.86 (−0.34 – 2.07)
		p = 0.01, r^2^ = 0.03	p = 0.16, r^2^ = 0.15	p = 0.16, r^2^ = 0.14
	Restless Leg Syndrome	−0.21 (−1.21 – 0.79)	−0.87 (−1.89 – 0.15)	−0.90 (−1.91 – 0.11)
		p = 0.68, r^2^ = 0.001	p = 0.09, r^2^ = 0.15	p = 0.08, r^2^ = 0.14

Finally, we dichotomized the participants based on the presence of m-TMD and re-ran the regression models to reveal the relationship of comorbidities and our outcome variables based on TMD subgroups. Tables 4 and 5 show that the associations in the combined TMD group held true for the m-TMD subgroup. However, Tables 6 and 7 indicate that in the j-TMD group, the only association observed was between the number of comorbidities and TMD pain duration. All other associations observed in the combined TMD group did not meet statistical significance in the j-TMD subgroup.

**Table 4:** Results of multiple linear regression models between comorbidities and TMD pain duration in participants in the m-TMD group (n = 121)

Dependent variables	Independent variables	Crude Model	Complex model	Simple model
		Coefficient (95 % CI)	Coefficient (95 % CI)	Coefficient (95 % CI)
		p value, r^2^ value	p value, r^2^ value	p value, r^2^ value
TMD duration (years)	Number of comorbidity	1.51 (0.35 – 2.66)	1.53 (0.33 – 2.73)	1.44 (0.25 – 2.62),
		p < 0.05, r^2^ = 0.05	p < 0.05, r^2^ = 0.07	p < 0.05, r^2^ = 0.11
	Migraine	1.11 (−1.72 – 3.94)	0.34 (−2.60 – 3.28)	−0.01 (−2.89 –2.87)
		p = 0.44, r^2^ = 0.01	p = 0.82, r^2^ = 0.18	p = 0.99, r^2^ = 0.17
	Chronic Fatigue Syndrome	5.38 (1.91 – 8.84),	5.98 (2.16 – 9.79),	5.54 (1.78 – 9.30),
		p < 0.01, r = 0.07	p < 0.01, r^2^ = 0.18	p < 0.01, r^2^ = 0.17
	Irritable Bowel Syndrome	3.08 (0.06 – 6.09)	3.14 (0.04 – 6.25)	3.14 (0.04 – 6.23)
		p = 0.05, r^2^ = 0.03	p = 0.05, r^2^ = 0.18	p = 0.05, r^2^ = 0.17
	Interstitial Cystitis	2.04 (−2.01 – 6.09)	−0.88 (5.17 – 3.42)	−0.71 (−4.98 – 3.56)
		p = 0.32, r^2^ = 0.01	p = 0.69, r^2^ = 0.18	p = 0.74, r^2^ = 0.17
	Restless Leg Syndrome	−0.23 (−4.03 – 3.56)	−1.74 (−5.54 – 2.07)	−1.69 (−5.47 – 2.09)
		p = 0.90, r^2^ = 0.001	p = 0.37, r^2^ = 0.18	p = 0.38, r^2^ = 0.17

**Table 5:** Results of multiple linear regression models between comorbidities and TMD pain intensity in participants in the m-TMD group (n = 121)

Dependent variables	Independent variables	Crude Model	Complex model	Simple model
		Coefficient (95 % CI)	Coefficient (95 % CI)	Coefficient (95 % CI)
		p value, r^2^ value	p value, r^2^ value	p value, r^2^ value
TMD intensity (0–10)	Number of comorbidity	0.63 (0.31 – 0.95),	0.60 (0.25 – 0.94),	0.62 (0.27 – 0.95),
		p < 0.01, r^2^ = 0.11	p < 0.01, r^2^ = 0.13	p < 0.01, r^2^ = 0.13
	Migraine	1.45 (0.64 – 2.25),	1.08 (0.21 – 1.95),	1.08 (0.23 – 1.93),
		p < 0.01, r^2^ = 0.10	p < 0.05, r^2^ = 0.18	p < 0.05, r^2^ = 0.18
	Chronic Fatigue Syndrome	1.82 (0.81 – 2.85),	1.42 (0.29 – 2.54),	1.42 (0.30 – 2.53),
		p < 0.01, r^2^ = 0.10	p < 0.05, r^2^ = 0.18	p < 0.05, r^2^ = 0.18
	Irritable Bowel Syndrome	0.36 (−0.56 – 1.27)	−0.05 (−0.97 – 0.87)	−0.05 (−0.97 – 0.87)
		p = 0.44, r^2^ = 0.01	p = 0.91, r^2^ = 0.18	p = 0.91, r^2^ = 0.18
	Interstitial Cystitis	1.36 (0.18 – 2.55)	0.71 (−0.56 – 1.98)	0.70 (−0.56 – 1.97)
		p = 0.03, r^2^ = 0.04	p = 0.27, r^2^ = 0.18	p = 0.27, r^2^ = 0.18
	Restless Leg Syndrome	0.61 (−0.51 – 1.74)	−0.12 (−1.25 – 0.99)	−0.13 (−1.24 – 0.99)
		p = 0.28, r^2^ = 0.01	p = 0.82, r^2^ = 0.18	p = 0.82, r^2^ = 0.18

**Table 6:** Results of multiple linear regression models between comorbidities and TMD pain duration in participants in the j-TMD group (n = 59)

Dependent variables	Independent variables	Crude Model	Complex model	Simple model
		Coefficient (95 % CI)	Coefficient (95 % CI)	Coefficient (95 % CI)
		p value, r^2^ value	p value, r^2^ value	p value, r^2^ value
TMD duration (years)	Number of comorbidity	2.77 (0.80 – 4.73),	2.54 (0.31 – 4.77),	2.38 (0.33 – 4.44),
		p < 0.01, r^2^ = 0.12	p < 0.05, r^2^ = 0.21	p < 0.05, r^2^ = 0.20
	Migraine	2.43 (−2.59 – 7.46)	0.05 (−5.83 – 5.93)	0.39 (−5.40 – 6.17)
		p = 0.34, r^2^ = 0.02	p = 0.99, r^2^ = 0.26	p = 0.89, r^2^ = 0.25
	Chronic Fatigue Syndrome	14.90 (5.23 – 24.55)	8.20 (−4.20 – 20.60),	9.89 (−1.71 – 21.48),
		p < 0.05, r^2^ = 0.14	p = 0.19, r^2^ = 0.26	p = 0.09, r^2^ = 0.25
	Irritable Bowel Syndrome	2.80 (−2.53 – 8.14)	0.69 (−5.36 – 6.74)	0.69 (4.96 – 6.34)
		p = 0.30, r^2^ = 0.02	p = 0.82, r^2^ = 0.26	p = 0.81, r^2^ = 0.25
	Interstitial Cystitis	10.20 (2.43 – 17.98)	5.58 (−4.99 –16.14)	3.64 (−6.05 – 13.33)
		p = 0.01, r^2^ = 0.11	p = 0.29, r^2^ = 0.26	p = 0.45, r^2^ = 0.25
	Restless Leg Syndrome	5.52 (−0.40 – 11.45)	2.86 (−3.99 – 9.70)	2.30 (−4.20 – 8.81)
		p = 0.07, r^2^ = 0.06	p = 0.41, r^2^ = 0.26	p = 0.48, r^2^ = 0.25

**Table 7:** Results of multiple linear regression models between comorbidities and TMD pain intensity in participants in the j-TMD group (n = 59)

Dependent variables	Independent variables	Crude Model	Complex model	Simple model
		Coefficient (95 % CI)	Coefficient (95 % CI)	Coefficient (95 % CI)
		p value, r^2^ value	p value, r^2^ value	p value, r^2^ value
TMD intensity (0–10)	Number of comorbidity	0.24 (−0.44 – 0.92)	0.20 (−0.57 – 0.97)	0.12 (−0.59 – 0.84)
		p = 0.49, r^2^ = 0.01	p = 0.60, r^2^ = 0.09	p = 0.73, r^2^ = 0.09
	Migraine	0.99 (−0.64 – 2.61)	0.34 (−1.62 – 2.32)	0.34 (−1.67 – 2.29)
		p = 0.23, r^2^ = 0.03	p = 0.73, r^2^ = 0.19	p = 0.73, r^2^ = 0.19
	Chronic Fatigue Syndrome	0.84 (−2.54 – 4.22)	0.08 (−3.94 – 4.09)	−0.08 (−4.00 – 3.82)
		p = 0.62, r^2^ = 0.004	p = 0.97, r^2^ = 0.19	p = 0.97, r^2^ = 0.19
	Irritable Bowel Syndrome	1.17 (−0.55 – 2.90)	0.75 (−1.29 – 2.79)	0.59 (−1.31 – 2.50)
		p = 0.18, r^2^ = 0.03	p = 0.47, r^2^ = 0.19	p = 0.53, r^2^ = 0.19
	Interstitial Cystitis	1.16 (−1.50 – 3.82)	1.65 (1.66 – 4.97)	1.73 (−1.53 – 5.01)
		p = 0.39, r^2^ = 0.01	p = 0.32, r^2^ = 0.19	p = 0.29, r^2^ = 0.19
	Restless Leg Syndrome	−1.85 (−3.77 – 0.08)	−2.13 (4.42 – 0.14)	−2.26 (−4.46 – −0.07)
		p = 0.06, r^2^ = 0.06	p = 0.07, r^2^ = 0.19	p = 0.04, r^2^ = 0.19

## Discussion

In this cross-sectional study, more than 62 % of participants suffered from at least one comorbid condition, with migraine, IBS and RLS being the most prevalent. This percentage is similar to previous results, showing that 70 % of people with painful TMD had at least one comorbidity [32]. Clearly, people with chronic TMD seeking treatment have a large overlap with other body pains. Compared to people without comorbidities, participants with comorbidities were more likely to be females with m-TMD, with a history of depression and/or anxiety and experiencing higher TMD pain intensity. These differences confirm earlier research, suggesting that the presence of comorbidities in patients with TMD may signify a more complex disorder [5, 7, 33–36].

Our study revealed a positive relationship between the number of comorbid conditions present and TMD pain duration and intensity. To our knowledge, our study is the first to look at this relationship using linear regression analyses, allowing control for other comorbid conditions and confounders. One previous study found a correlation between increasing number of pain symptoms and greater TMD pain severity [37], while other studies have found higher TMD pain intensity in subjects with widespread pain, compared to subjects with localized pain [7, 38]. No previous study has looked at the association between TMD pain duration and increasing number of pain sites. However, previous studies exist looking at persistent TMD pain, which could be comparable to long duration of TMD pain. For example, a longitudinal study revealed that the number of bodily pain sites were positively associated with having a persistent TMD pain (pain after 5 years), compared to TMD patients in remission (no pain after 5 years), with odds of 1.81 [11]. Other studies showed that baseline widespread pain was the most predictive factor for persistent TMD pain (OR = 1.78 - 1.99) [6, 39].

The relationship between the presence of comorbidities and TMD pain duration and intensity appear to be related to central sensitization [40, 41]. TMD patients with comorbidities have evidence of central sensitization and allodynia [42, 43], which may explain the increased TMD pain duration and intensity. As well, chronic TMD is associated with neural abnormalities in the trigeminal and limbic systems, which may be related to spreading of pain to other body parts [44].

The results show that the presence of migraine was associated with an increased TMD intensity. Migraine is prevalent among TMD patients, with a prevalence of migraine 22–58 % [45–47] and it is associated with increased physical and psychosocial disability [14–17]. The association between migraine and increased TMD pain intensity has been observed in a prior study, where the relative risk of having moderate/severe TMD compared to mild TMD with migraine comorbidity was 7.8 [47]. Furthermore, an association between frequency of headaches and TMD pain duration and intensity has been observed [48]. We also show that CFS is associated with TMD pain intensity and duration. The association between CFS and TMD pain duration was exceptionally high, suggesting a more profound central sensitization [49]. CFS is a condition that can overlap with TMD (20 %) [50]. However, fibromyalgia (FM), a condition that overlaps with both TMD and CFS was not controlled for in our study [49]. TMD patients with FM experience more intense and prolonged pain than TMD patients without FM [49]. Therefore, it is possible that the influence of CFS on TMD pain intensity and duration was partially or fully mediated by the presence of FM. The other conditions, IBS, IC and RLS were not associated with either TMD pain intensity or duration. However, such association could have been found with a larger sample size; an ad-hoc sample size calculation revealed that the sample size would need to be 333 to statistically observe an association if the effect size was small (f^2^ = 0.05), based on the numbers of predictors that were used in the current study.

This study also shows a difference between the m-TMD and j-TMD groups, where the j-TMD group did not display strong associations between most outcome and exposure variables. This may support the notion that j-TMD disease does not involve central sensitization [51–53]. However, an association between migraine and both m-TMD and j-TMD has been previously observed [54]. Since the sample size of j-TMD group was smaller than the m-TMD group (n = 59 vs. 121, respectively), the study may have been underpowered. Specifically, the regression results between CFS and TMD pain duration suggest that an association may exist but was not adequately detected.

This study has some limitations. Firstly, being a cross-sectional study, it cannot provide information regarding the direction of association between comorbidities and pain characteristics in TMD patients. Secondly, the subjects were people seeking treatment at a hospital center for TMD. They may not represent the average profile of a TMD patient, which has been shown to have acute, self-limiting pain [55]. Therefore, our study results could be more generalizable for people with chronic TMD seeking treatment. Thirdly, the study focused on the impact of specific comorbidities on TMD pain characteristics without taking into account the intensity or duration of these disorders. Other possible limitations include relatively small sample size that could result in missing certain associations in the final analysis, measurement errors, particularly for pain duration. Finally, a measurement bias may exist since emotional distress and psychological status may not have been adequately assessed and controlled for all subjects [56]. To assess psychological variables, patients were asked about a history of depression and anxiety, responding with a simple yes/no answer. A more accurate assessment could be done using a validated surveys like the Hospital Anxiety and Depression Scale [57].

## Conclusions

This study shows that in people with chronic TMD, increasing number of comorbid conditions was positively associated with TMD pain duration and intensity. Also, the presence of some conditions, such as migraine and CFS, are associated with increased TMD intensity and duration. These findings could be clinically relevant, suggesting that in order to improve TMD symptoms other existing comorbidities need to be assessed and addressed, probably in interdisciplinary clinics hosting multiple health professions [55].

## Abbreviations

TMD: Temporomandibular disorder
CFS: Chronic fatigue syndrome
RLS: Restless leg syndrome
IC: Interstitial cystitis
FM: Fibromyalgia
VIF: variance inflation factor
m-TMD: Myofascial TMD
j-TMD: Non-myofascial TMD

## References

[1] Rollman GB, Gillespie JM (2000). The role of psychosocial factors in temporomandibular disorders. Curr Rev Pain.

[2] Magnusson T, Egermarki I, Carlsson GE (2005). A prospective investigation over two decades on signs and symptoms of temporomandibular disorders and associated variables. A final summary. Acta Odontol Scand.

[3] Clark GT (1987). Diagnosis and treatment of painful temporomandibular disorders. Dent Clin N Am.

[4] Hoffmann RG, Kotchen JM, Kotchen TA, Cowley T, Dasgupta M, Cowley AW (2011). Temporomandibular disorders and associated clinical comorbidities. Clin J Pain.

[5] de Leeuw R, Klasser GD, Albuquerque RJ (2005). Are female patients with orofacial pain medically compromised?. J Am Dent Assoc.

[6] Velly AM, Look JO, Carlson C, Lenton PA, Kang W, Holcroft CA, Fricton JR (2011). The effect of catastrophizing and depression on chronic pain–a prospective cohort study of temporomandibular muscle and joint pain disorders. Pain.

[7] Velly AM, Look JO, Schiffman E, Lenton PA, Kang WJ, Messner RP, Holcroft CA, Fricton JR (2010). The effect of Fibromyalgia and widespread pain on the clinically significant temporomandibular muscle and joint pain disorders-a prospective 18-month Cohort study. Journal of Pain.

[8] Turp JC, Kowalski CJ, O'Leary N, Stohler CS (1998). Pain maps from facial pain patients indicate a broad pain geography. J Dent Res.

[9] John MT, Miglioretti DL, LeResche L, Von Korff M, Critchlow CW (2003) Widespread pain as a risk factor for dysfunctional temporomandibular disorder pain. Pain 102 (3):257–263. doi:10.1016/s0304-3959(02)00404-9 10.1016/S0304-3959(02)00404-9 12670667

[10] Bair E, Ohrbach R, Fillingim RB, Greenspan JD, Dubner R, Diatchenko L, Helgeson E, Knott C, Maixner W, Slade GD (2013). Multivariable modeling of phenotypic risk factors for first-onset TMD: the OPPERA prospective cohort study. The journal of pain: official journal of the American Pain Society.

[11] Rammelsberg P, Leresche L, Dworkin S, Mancl L (2003). Longitudinal outcome of temporomandibular disorders: a 5-year epidemiologic study of muscle disorders defined by research diagnostic criteria for temporomandibular disorders. J Orofac Pain.

[12] Natvig B, Bruusgaard D, Eriksen W (2001). Localized low back pain and low back pain as part of widespread musculoskeletal pain: two different disorders? A cross-sectional population study. Journal of rehabilitation medicine: official journal of the UEMS European Board of Physical and Rehabilitation Medicine.

[13] Singh JA, Lewallen DG (2013). Medical and psychological comorbidity predicts poor pain outcomes after total knee arthroplasty. Rheumatology.

[14] Fragoso YD, Alves HH, Garcia SO, Finkelsztejn A (2010). Prevalence of parafunctional habits and temporomandibular dysfunction symptoms in patients attending a tertiary headache clinic. Arq Neuropsiquiatr.

[15] da Silva A, Jr., Costa EC, Gomes JB, Leite FM, Gomez RS, Vasconcelos LP, Krymchantowski A, Moreira P, Teixeira AL (2010) Chronic headache and comorbidities: a two-phase, population-based, cross-sectional study. Headache 50 (8):1306–1312. doi:10.1111/j.1526-4610.2010.01620.x 10.1111/j.1526-4610.2010.01620.x 20163479

[16] Ballegaard V, Thede-Schmidt-Hansen P, Svensson P, Jensen R (2008). Are headache and temporomandibular disorders related? A blinded study. Cephalalgia: an international journal of headache.

[17] Mitrirattanakul S, Merrill RL (2006). Headache impact in patients with orofacial pain. J Am Dent Assoc.

[18] Auerbach SM, Laskin DM, Frantsve LM, Orr T (2001). Depression, pain, exposure to stressful life events, and long-term outcomes in temporomandibular disorder patients. Journal of oral and maxillofacial surgery: official journal of the American Association of Oral and Maxillofacial Surgeons.

[19] Turk DC, Rudy TE (1987). IASP taxonomy of chronic pain syndromes: preliminary assessment of reliability. Pain.

[20] Dworkin SF, Leresche L (1992). Research diagnostic criteria for temporomandibular disorders: review, criteria, examinations and specifications, critique. Journal of craniomandibular disorders: facial & oral pain.

[21] Viera AJ, Garrett JM (2005). Understanding interobserver agreement: the kappa statistic. Fam Med.

[22] Kelley K, Maxwell SE (2003). Sample size for multiple regression: obtaining regression coefficients that are accurate, not simply significant. Psychol Methods.

[23] Lipton RB, Dodick D, Sadovsky R, Kolodner K, Endicott J, Hettiarachchi J, Harrison W (2003). A self-administered screener for migraine in primary care: the ID Migraine validation study. Neurology.

[24] Hadzi-Pavlovic D, Hickie IB, Wilson AJ, Davenport TA, Lloyd AR, Wakefield D (2000). Screening for prolonged fatigue syndromes: validation of the SOFA scale. Soc Psychiatry Psychiatr Epidemiol.

[25] Whitehead WE, Drossman DA (2010) Validation of symptom-based diagnostic criteria for irritable bowel syndrome: a critical review. The American journal of gastroenterology 105 (4):814–820; quiz 813, 821. doi:10.1038/ajg.2010.56. 10.1038/ajg.2010.56 PMC3856202 20179688

[26] Kushner L, Moldwin RM (2006) Efficiency of questionnaires used to screen for interstitial cystitis. The Journal of urology 176 (2):587–592. doi:10.1016/j.juro.2006.03.035. 10.1016/j.juro.2006.03.035 16813894

[27] Allen RP, Burchell BJ, MacDonald B, Hening WA, Earley CJ (2009) Validation of the self-completed Cambridge-Hopkins questionnaire (CH-RLSq) for ascertainment of restless legs syndrome (RLS) in a population survey. Sleep medicine 10 (10):1097–1100. doi:10.1016/j.sleep.2008.10.007 10.1016/j.sleep.2008.10.007 19195928

[28] Mccaffery M, Beebe A (1989). Pain: Clinical Manual for nursing practice.

[29] Hosmer DW, Lemenshow, S., Sturdivant, R.X. (2013) Applied Logistic Regression, vol Third Edition. John Wiley & Sons

[30] Lee PH (2014). Is a cutoff of 10 % appropriate for the change-in-estimate criterion of confounder identification?. Journal of epidemiology/Japan Epidemiological Association.

[31] Fillingim RB, Ohrbach R, Greenspan JD, Knott C, Dubner R, Bair E, Baraian C, Slade GD, Maixner W (2011). Potential psychosocial risk factors for chronic TMD: descriptive data and empirically identified domains from the OPPERA case–control study. The journal of pain: official journal of the American Pain Society.

[32] Ohrbach R, Fillingim RB, Mulkey F, Gonzalez Y, Gordon S, Gremillion H, Lim PF, Ribeiro-Dasilva M, Greenspan JD, Knott C, Maixner W, Slade G (2011). Clinical findings and pain symptoms as potential risk factors for chronic TMD: descriptive data and empirically identified domains from the OPPERA case–control study. The journal of pain: official journal of the American Pain Society.

[33] Lorduy KM, Liegey-Dougall A, Haggard R, Sanders CN, Gatchel RJ (2013). The prevalence of comorbid symptoms of central sensitization syndrome among three different groups of temporomandibular disorder patients. Pain practice: the official journal of World Institute of Pain.

[34] Yunus MB (2009). Central sensitivity syndromes: an overview. J Musculoskeletal Pain.

[35] Velly AM, Fricton J (2011). PAIN UPDATE The impact of comorbid conditions on treatment of temporomandibular disorders. J Am Dent Assoc.

[36] Velly AM, Gornitsky M, Philippe P (2003). Contributing factors to chronic myofascial pain: a case–control study. Pain.

[37] Yap AU, Chua EK, Dworkin SF, Tan HH, Tan KB (2002). Multiple pains and psychosocial functioning/psychologic distress in TMD patients. Int J Prosthodont.

[38] Raphael KG, Marbach JJ, Klausner J (2000). Myofascial face pain - Clinical characteristics of those with regional vs. widespread pain. J Am Dent Assoc.

[39] Macfarlane TV, Blinkhorn AS, Davies RM, Kincey J, Worthington HV (2004) Predictors of outcome for orofacial pain in the general population: a four-year follow-up study. Journal of dental research 83 (9):712–717. 10.1177/154405910408300911 15329378

[40] Franco AL, Fernandes G, Goncalves DA, Bonafe FS, Camparis CM (2014). Headache associated with temporomandibular disorders among young Brazilian adolescents. Clin J Pain.

[41] Woolf CJ (2011). Central sensitization: implications for the diagnosis and treatment of pain. Pain.

[42] Bevilaqua-Grossi D, Lipton RB, Napchan U, Grosberg B, Ashina S, Bigal ME (2010). Temporomandibular disorders and cutaneous allodynia are associated in individuals with migraine. Cephalalgia: an international journal of headache.

[43] Grossi DB, Lipton RB, Bigal ME (2009). Temporomandibular disorders and migraine chronification. Curr Pain Headache Rep.

[44] Younger JW, Shen YF, Goddard G, Mackey SC (2010). Chronic myofascial temporomandibular pain is associated with neural abnormalities in the trigeminal and limbic systems. Pain.

[45] Franco AL, Goncalves DAG, Castanharo SM, Speciali JG, Bigal ME, Camparis CM (2010). Migraine is the Most Prevalent Primary Headache in Individuals with Temporomandibular Disorders. J Orofac Pain.

[46] Kang JK, Ryu JW, Choi JH, Merrill RL, Kim ST (2010) Application of ICHD-II criteria for headaches in a TMJ and orofacial pain clinic. Cephalalgia : an international journal of headache 30 (1):37–41. doi:10.1111/j.1468-2982.2009.01866.x. 10.1111/j.1468-2982.2009.01866.x 19438924

[47] Goncalves DA, Camparis CM, Speciali JG, Franco AL, Castanharo SM, Bigal ME (2011). Temporomandibular disorders are differentially associated with headache diagnoses: a controlled study. Clin J Pain.

[48] Anderson GC, John MT, Ohrbach R, Nixdorf DR, Schiffman EL, Truelove ES (2011). Influence of headache frequency on clinical signs and symptoms of TMD in subjects with temple headache and TMD pain. Pain.

[49] Pfau DB, Rolke R, Nickel R, Treede RD, Daublaender M (2009). Somatosensory profiles in subgroups of patients with myogenic temporomandibular disorders and fibromyalgia syndrome. Pain.

[50] Aaron LA, Burke MM, Buchwald D (2000). Overlapping conditions among patients with chronic fatigue syndrome, fibromyalgia, and temporomandibular disorder. Arch Intern Med.

[51] Klasser GD, Bassiur J, Leeuw R (2014). Differences in reported medical conditions between myogenous and arthrogenous TMD patients and its relevance to the general practitioner. Quintessence Int.

[52] Galdon MJ, Dura E, Andreu Y, Ferrando M, Poveda R, Bagan JV (2006). Multidimensional approach to the differences between muscular and articular temporomandibular patients: Coping, distress, and pain characteristics. Oral Surg Oral Med Oral Pathol Oral Radiol Endod.

[53] Yunus MB (2008). Central sensitivity syndromes: a new paradigm and group nosology for fibromyalgia and overlapping conditions, and the related issue of disease versus illness. Semin Arthritis Rheum.

[54] Goncalves MC, Florencio LL, Chaves TC, Speciali JG, Bigal ME, Bevilaqua-Grossi D (2013). Do women with migraine have higher prevalence of temporomandibular disorders?. Brazilian journal of physical therapy.

[55] Velly AM, Fricton J (2011). The impact of comorbid conditions on treatment of temporomandibular disorders. J Am Dent Assoc.

[56] Pannucci CJ, Wilkins EG (2010). Identifying and avoiding bias in research. Plast Reconstr Surg.

[57] Zigmond AS, Snaith RP (1983). The hospital anxiety and depression scale. Acta Psychiatr Scand.

